# Complete genome sequence of a *Listeria monocytogenes* serotype 1 isolated from a critically ill patient in Italy, 2023

**DOI:** 10.1128/mra.01240-23

**Published:** 2024-06-12

**Authors:** Stefano Amadesi, Caterina Vocale, Davide Guariglia, Tiziana Lazzarotto, Simone Ambretti, Paolo Gaibani

**Affiliations:** 1 Microbiology Unit, IRCCS Azienda Ospedaliero-Universitaria di Bologna, Bologna, Italy; 2 Department of Medical and Surgical Sciences, Alma Mater Studiorum, University of Bologna, Bologna, Italy; 3 Department of Diagnostic and Public Health, Microbiology Section, Verona University, Verona, Italy; University of Maryland School of Medicine, Baltimore, Maryland, USA

**Keywords:** *Listeria monocytogenes*, invasive bacteria infection, bloodstream infections

## Abstract

*Listeria monocytogenes*, a concerning foodborne pathogen, causes severe infections in vulnerable subjects such as pregnant women and the elderly. In this article, we present the complete genome sequence of P4_LIS, an *L. monocytogenes* isolated from a patient with invasive bacteria infection.

## ANNOUNCEMENT

*Listeria monocytogenes* poses a significant food safety threat (1). Upon ingestion of contaminated food, *L. monocytogenes* can induce neonatal listeriosis in pregnant women and meningitis in older individuals (2). Outbreaks are commonly associated with serotype 4b, while serotypes 1/2a, 1/2b, and 1/2 c are linked to sporadic food intoxications (3). Here, we present P4_LIS, an *L. monocytogenes* strain belonging to serotype 1 responsible for meningitis and bacteremia in a patient with cancer. A blood sample was inoculated on chocolate agar and incubated for 24 hours at 37°C. Colonies were observed and confirmed as gram-positive by Gram staining. Positive colonies were identified as *L. monocytogenes* using the MALDI Biotyper (Bruker, Massachusetts, USA) system following a standard diagnostic procedure (4). A single colony was cultured on a Blood Agar plate overnight at 37°C to obtain a pure bacterial culture. DNA was extracted using the Dneasy Blood and Tissue Kit (Qiagen, Germany), purified with AMPure XP Bead-Based Reagent (Beckman Coulter, California, USA), and quantified using the Qubit double-stranded DNA(dsDNA) Quantification BR Assay Kit (Thermo Fisher, Massachusetts, USA). Illumina libraries were prepared with the Illumina DNA Prep Kit (Illumina, USA), while the Nanopore Rapid Sequencing Kit V14 (SQK-RAD114; Oxford Nanopore Technologies, UK) was used for Nanopore libraries. No shearing or size selection was performed. Sequencing was performed using the Illumina iSeq 100 and Nanopore MinION platforms. For Nanopore reads, basecalling was performed locally with MinKNOW v22.12.5 (https://community.nanoporetech.com/docs/sequence/sequencing_software/minknow-tech-doc/v/mitd_5000_v1_revaj_16may2016). Read quality was evaluated with FastQC v0.12.1 (5). No trimming was performed. The genome was assembled with Unicycler v0.5.0 (6), circularized, and rotated setting *dnaA* as starting position using Circlator v1.5.5 (7) (parameters --merge_min_id 85 and --merge_breaklen 1000). Circularity was verified with Bandage v0.8.1 (8). Polishing was performed with short reads using Polypolish v0.5.0 (9). Sequence type was determined with MLST v2.23.0 (https://github.com/tseemann/mlst), while serotype was predicted using LisSero v0.4.9 (https://github.com/MDU-PHL/LisSero) (10). The genome was annotated with PGAP v6.6 (11) and scanned for antibiotic resistance and virulence genes using Abricate v1.0.1 (https://github.com/tseemann/abricate) and BLAST v2.14.0. Default parameters were used for all software unless specified. Illumina sequencing produced 2,140,587 reads, 151 bp in length. Nanopore sequencing had an output of 100,549 reads with an average length of 2,592 bp, ranging from a length of 97 to 85,375 bp, 6,543 N50. The genome assembly was composed of a single, circular contig 3,027,625 bp in length, with 38.01% G+C content and 193× mean coverage. Typing revealed that isolate P4_LIS belonged to sequence type ST9, serogroup I.2 (serotype 1/2 c-3c). Sequence analysis revealed that the isolate carried genes potentially involved in resistance against fosfomycin (*fosX*), lincosamides (*lin*), cationic peptides (*mprF*), and encoding for multi-drug efflux pumps (*mdrL* and *norB*). Moreover, putative virulence factors were detected, including listeriolysin (*hly*), internalins (*inlA*, *inlB*, *inlC*, *inlF*, *inlH*, *inlJ*, and *inlK*), secreted enzymes (*mpl*, *plcA*, and *plcB*), chaperones (*prsA1* and *prsA2*), enzymes involved in regulation (*prfA*), invasion (*aut*, *iap*/*cwhA*, and *ipeA*), protein maturation (*lspA* and *lspB*), metabolic adaptation (*hpt*, *lplA1*, and *lplA2*), adhesion (*ami*, *fbpA*, *gtcA*, *lap*, *lapB*, and *vip*), actin assembly (*actA*), stress response (*bsh*, *clpB*, *clpC*, *clpE*, and *clpP*), and immune response modulation (*lntA*, *oatA*, and *pdgA*).

## Data Availability

The complete genome assembly of P4_LIS strain has been deposited in the NCBI GenBank database under accession number CP139336.1. Sequencing reads have been deposited in the NCBI SRA database under accession numbers SRR27184774 and SRR27184773.
